# Dual-isotope imaging allows in vivo immunohistochemistry using radiolabelled antibodies in tumours^☆^^☆☆^

**DOI:** 10.1016/j.nucmedbio.2019.01.010

**Published:** 2019-03

**Authors:** James C. Knight, Michael J. Mosley, Veerle Kersemans, Gemma M. Dias, P. Danny Allen, Sean Smart, Bart Cornelissen

**Affiliations:** CRUK/MRC Oxford Institute for Radiation Oncology, Department of Oncology, University of Oxford, Oxford, United Kingdom

**Keywords:** Dual-isotope, HER2, PET, SPECT, Molecular imaging, Antibody

## Abstract

While radiolabelled antibodies have found great utility as PET and SPECT imaging agents in oncological investigations, a notable shortcoming of these agents is their propensity to accumulate non-specifically within tumour tissue. The degree of this non-specific contribution to overall tumour uptake is highly variable and can ultimately lead to false conclusions. Therefore, in an effort to obtain a reliable measure of inter-individual differences in non-specific tumour uptake of radiolabelled antibodies, we demonstrate that the use of dual-isotope imaging overcomes this issue, enables true quantification of epitope expression levels, and allows non-invasive in vivo immunohistochemistry. The approach involves co-administration of (i) an antigen-targeting antibody labelled with zirconium-89 (^89^Zr), and (ii) an isotype-matched non-specific control IgG antibody labelled with indium-111 (^111^In). As an example, the anti-HER2 antibody trastuzumab was radiolabelled with ^89^Zr, and co-administered intravenously together with its ^111^In-labelled non-specific counterpart to mice bearing human breast cancer xenografts with differing HER2 expression levels (MDA-MB-468 [HER2-negative], MDA-MB-231 [low-HER2], MDA-MB-231/H2N [medium-HER2], and SKBR3 [high-HER2]). Simultaneous PET/SPECT imaging using a MILabs Vector4 small animal scanner revealed stark differences in the intratumoural distribution of [^89^Zr]Zr-trastuzumab and [^111^In]In-IgG, highlighting regions of HER2-mediated uptake and non-specific uptake, respectively. Normalisation of the tumour uptake values and tumour-to-blood ratios obtained with [^89^Zr]Zr-trastuzumab against those obtained with [^111^In]In-IgG yielded values which were most strongly correlated (R = 0.94; P = 0.02) with HER2 expression levels for each breast cancer type determined by Western blot and in vitro saturation binding assays, but not non-normalised uptake values. Normalised intratumoural distribution of [^89^Zr]Zr-trastuzumab correlated well with intratumoural heterogeneity HER2 expression.

## 1 Introduction

Nuclear imaging techniques such as positron emission tomography (PET) and single-photon emission computed tomography (SPECT) often utilise radiolabelled antibodies to visualise cancer-associated antigens located within malignant tumours [[1], [2], [3], [4], [5], [6]]. Antibodies can offer extremely high binding affinities and specificities towards their target antigens. Therefore, radiolabelled antibodies are an excellent choice for non-invasive imaging and monitoring changes of those target antigens over a time course, e.g. to monitor cancer treatment. Ideally, the accumulation of the antibody imaging agent within tumours would be mediated entirely by the relevant target antigen. However, complications invariably arise when non-specific phenomena contribute to their overall tumour uptake. One example of such non-specific factors is the enhanced permeability and retention (EPR) effect, which stems from rapid and irregular angiogenesis and causes antibodies to passively extravasate to tumour tissue via the newly formed ‘leaky vasculature’ [[7], [8], [9]]. It is also recognised that the necrotic areas that develop within poorly vascularised tumours can further influence the distribution of pharmaceutical agents within tumours [10]. These non-specific contributions to overall tumour uptake can vary wildly between tumour models, within one single tumour (intra-tumoural heterogeneity) or as a result of differential response to treatment (inter-tumoural heterogeneity). This may reduce the sensitivity of these imaging techniques and make false discoveries more likely [11].

Therefore, the ability to obtain an accurate measure of only specific tumour uptake (i.e. any uptake directly mediated by the target antigen) would allow a more informed and meaningful assessment of each imaging investigation. Clearly, this would significantly benefit any basic and pre-clinical research investigations involving radiolabelled antibodies in animal models of cancer. At the same time, the technique improves statistical analysis of results, while halving the number of animals needed to come to any conclusion.

With these aims in mind, we applied dual-isotope imaging, based on co-administration of an antigen-targeting antibody (in this case, trastuzumab) and an isotype-matched non-specific antibody (IgG1/κ). These antibodies were radiolabelled with zirconium-89 and indium-111, respectively, with distinctly different gamma emission spectra, which allows their biodistribution profiles to be tracked individually. This was accomplished using a MILabs Vector4 SPECT/CT system with energy-resolved detectors and by performing image reconstructions based on the unique γ-emission energies of each radioisotope.

Multi-isotope SPECT or SPECT/PET imaging techniques have certainly previously been utilised in angiogenesis [12], brain [13], cardiac [14], infection [15,16], and thrombus [17] imaging investigations, including in the clinic. The most common radioisotope combinations used in these studies are ^111^In/^99m^Tc [18], ^123^I/^99m^Tc [13], ^131^I/^99m^Tc [19], ^201^Tl/^99m^Tc [20], ^111^In/^177^Lu [12], and ^125^I/^111^In/^68^Ga [17]. The combination of radioisotopes used here ^111^In/^89^Zr, is remarkably well-suited to dual-isotope imaging as the principle γ-emissions resulting from the decay of ^89^Zr at 511 keV (*β*^+^/*β*^−^ annihilation) and 909 keV are easily resolved from the lower energy γ-emissions of ^111^In (171 and 245 keV), resulting in minimal spectral overlap and crosstalk effects. Furthermore, the ^89^Zr and ^111^In radioisotopes also have well-matched physical half-lives of 3.3 and 2.8 days, respectively, which renders each of them compatible with the well-characterised pharmacokinetics of antibody agents.

The purpose of the current study was to evaluate dual-isotope antibody imaging in terms of its ability to provide an accurate and personalised measure of specific tumour uptake and thus improve quantification of antibody-based nuclear imaging. Furthermore, we investigated the use of dual-isotope imaging using radiolabelled antibodies to probe intratumoural epitope heterogeneity. To test this, ^89^Zr-labelled anti-HER2 antibody trastuzumab and its non-specific ^111^In-labelled counterpart were co-administered to mice bearing one of four human breast cancer xenografts with varying HER2 expression levels. The overall uptake of each radiolabelled agent within the tumours and relevant ratiometric values were subjected to correlational analysis with empirically-determined HER2-expression levels. At the same time, non-specific binding-corrected in vivo images were correlated to intratumourally heterogeneous HER2 expression levels.

## 2 Methods and materials

### 2.1 General methods

All reagents were purchased from Sigma-Aldrich unless otherwise stated and were used without further purification. The chelating agents *p*-SCN-Bn-DFO and *p*-SCN-Bn-DTPA were purchased from Macrocyclics Inc. (Dallas, TX). Water was deionised using a Barnstead NANOpure purification system (Thermo Scientific) and had a resistance of >18.2 MΩ cm^−1^ at 25 °C. Protein concentration measurements were made on a ND-1000 spectrophotometer (NanoDrop Technologies, Inc.). Instant thin-layer chromatography (iTLC) was performed on glass microfiber chromatography paper (Agilent Technologies) and strips were analysed with a Bioscan AR-2000 radio-TLC scanner (Eckert & Ziegler). pH was determined using pH indicator paper (Merck Millipore). Radioactivity measurements were made using a CRC-25R dose calibrator (Capintec, Inc.).

### 2.2 Cell culture

Four human breast cancer cell lines with differing expression levels of the cancer biomarker HER2 were used in this study: MDA-MB-468 [HER2-negative], MDA-MB-231 [low-HER2], MDA-MB-231/H2N [medium-HER2], and SKBR3 [high-HER2]. MDA-MB-231/H2N cells were obtained by stable transfection of the HER2 receptor in MDA-MB-231 cells [21]. MDA-MB-231/H2N cells were a kind gift from Professor Robert Kerbel at Sunnybrook Hospital, Toronto, Canada. All cell lines were maintained in Dulbecco's Modified Eagle Medium (DMEM), supplemented with 10% foetal bovine serum (FBS), 2 mM l-glutamine, 100 units/mL penicillin, and 0.1 mg mL^−1^ streptomycin. Cells were grown in a 37 °C environment containing 5% CO_2_ and were harvested and passaged as required using Trypsin-EDTA solution. The cumulative length of culture was <6 months following retrieval from liquid nitrogen storage.

### 2.3 Preparation of radiolabelled antibodies

Radiolabelling of trastuzumab and IgG was performed using previous described methods involving zirconium-89 [22] and indium-111 [23]. Radiolabelling yield (the amount of radionuclide incorporated into the final product versus the starting amount) was routinely >95%, as determined by iTLC-SG with 50 mM DTPA as the mobile phase. The final radiochemical purity of the radiolabelled antibodies (radionuclide associated with antibody versus non-associated radionuclide) after size exclusion chromatography was >99%.

The immunoreactivity of [^89^Zr]Zr-trastuzumab, the main imaging agent used for in vivo imaging, was determined on MDA-MB-231/H2N cells by linear extrapolation to conditions representing infinite antigen excess according to the method described by Lindmo et al. [24,25].

### 2.4 In vitro saturation binding studies

Aliquots of 2 × 10^5^ cells were seeded in 24-well plates in warm culture medium (500 μL) and were allowed to adhere. The supernatant was removed from the wells and was replaced by a serial dilution of radiolabelled trastuzumab (0.3 MBq/μg; concentrations as indicated) in phosphate buffered saline (PBS; pH 7.4; 500 μL). The cells were then incubated at 4 °C for 2 h. The supernatant was removed and the wells were washed twice with PBS (500 μL). Cells were then lysed using a solution of 0.1 N NaOH (250 μL) for 30 min at room temperature. The resulting lysates were transferred to counting tubes, combined with two further washes of 0.1 N NaOH, and the amount of radioactivity was measured using a 2480 WIZARD^2^ gamma counter (PerkinElmer).

### 2.5 In vivo studies

All animal procedures were performed in accordance with the UK Animals (Scientific Procedures) Act 1986 and with local ethical committee approval. Xenograft tumours were established in the right hind flank of female BALB/c *nu*/*nu* mice (Harlan) by subcutaneous injection of 5 × 10^6^ cells in a 1:1 mixture of fresh media and BD Matrigel basement membrane matrix (BD Biosciences) (100 μL).

### 2.6 Dual-isotope imaging experiments

When tumours reached a size of approximately 180 mm^3^, mice were administered a co-injection of [^89^Zr]Zr-Trastuzumab (0.9 ± 0.2 MBq, 10 μg) and [^111^In]In-IgG (3.9 ± 0.8 MBq, 10 μg) in sterile phosphate buffered saline (100 μL) intravenously via the lateral tail vein. PET/SPECT/CT images were acquired using a VECTor^4^CT scanner (MILabs) at 72 h after injection. As a control, some mice were administered a co-injection of [^111^In]In-Trastuzumab (4 MBq, 10 μg) and [^89^Zr]Zr-IgG (1 MBq, 10 μg). For full experimental details, including acquisition and reconstruction parameters, see Supporting Information. The ability of the imaging system to simultaneously acquire images for ^111^In and ^89^Zr was evaluated using phantoms containing mixtures of known amounts of either radionuclide (Fig. S1). Briefly, a series of five phantoms were used, phantoms 1 and 5 contained only ^111^In or ^89^Zr respectively. Phantoms 2–4 contained a mixture of ^89^Zr and ^111^In. Images were obtained of these phantoms with the same imaging parameters and reconstruction parameters as the in vivo studies.

### 2.7 Ex vivo biodistribution experiments

Mice were euthanized by cervical dislocation and selected tissues and blood were removed, immediately rinsed with water, blot dried, and transferred into a pre-weighed counting tube. The amount of ^111^In and ^89^Zr in each sample was measured simultaneously using a HiDex gamma counter (Lablogic). After correction for downscatter, counts per minute were converted into radioactivity units (MBq) using calibration curves generated from known standards. These values were decay-corrected to the time of injection, and the percentage of the injected dose per gram (%ID/g) of each sample was calculated.

### 2.8 Western blot

Tumour xenograft tissues were used to prepare total cell lysates. Lysates were separated by SDS-PAGE, and transferred to PVDF membrane. Membranes were blocked, and stained for HER-2 using a rabbit monoclonal antibody (Cell Signalling Technology 29D8-2165; 1:1000 dilution), and visualised using a horseradish peroxidase secondary antibody (Thermo Scientific 65-6120; 1:5000). For full experimental details, see Supporting Information.

### 2.9 Statistical analyses

All statistical analyses and nonlinear regression were performed using GraphPad Prism version 7 (GraphPad Software, San Diego, CA, USA). Data were tested for normality using a Shapiro-Wilk test. Analysis of variance (ANOVA) with Holm-Sidak's post-test was used to calculate the significance of differences between groups. All data were obtained at least in triplicate and results reported as mean ± standard deviation, unless stated otherwise.

## 3 Results

### 3.1 In vitro analyses

**Fig. 1:**
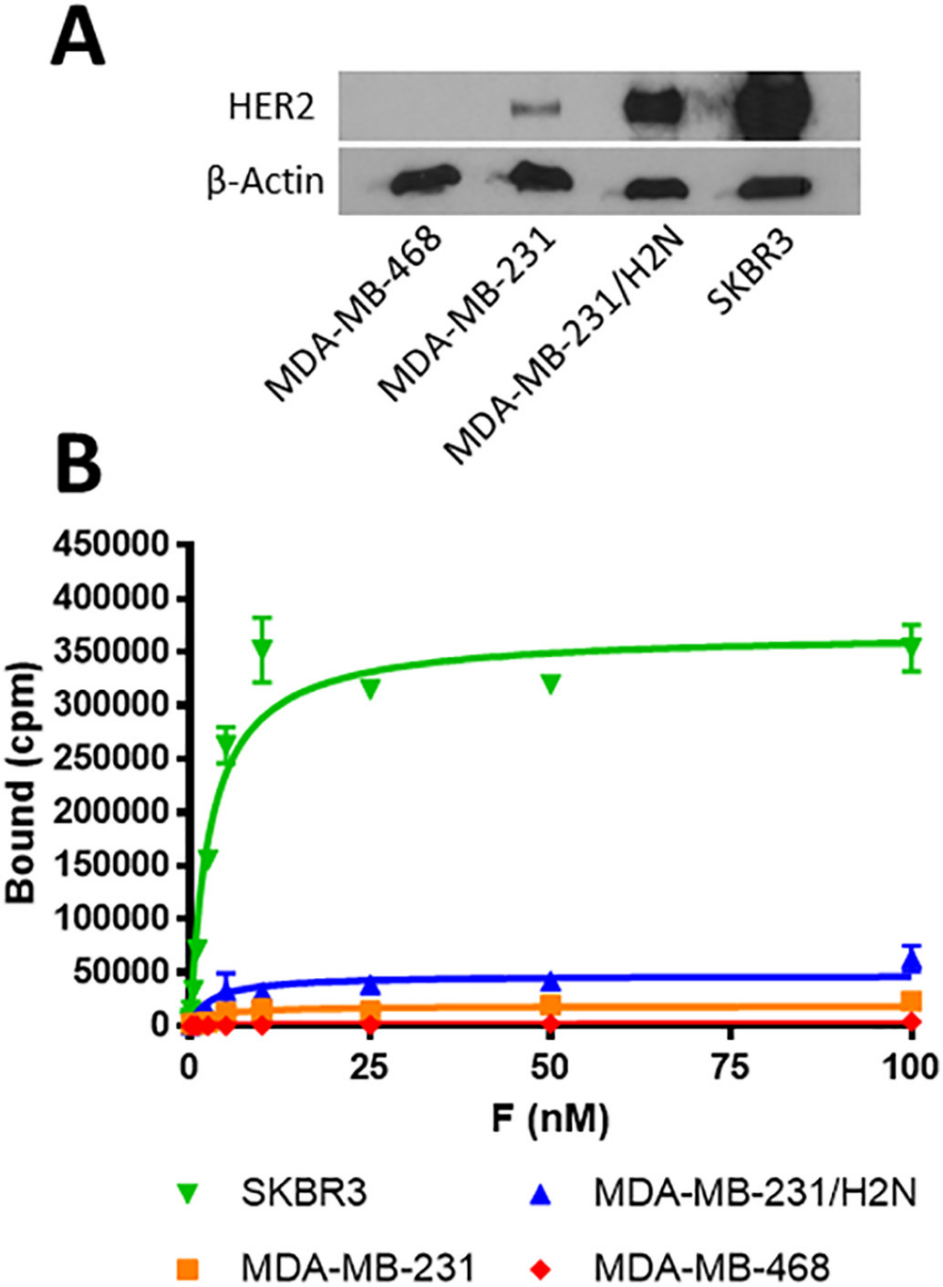
(A) Western blot of tumour xenograft lysates showing the different levels of HER2 expression. (B) Saturation binding plot of [^111^In]In-trastuzumab on each of the cell lines used in the study.

Relative HER2 expression levels in xenograft tumours established from each of the four breast cancer cell lines was visualised and quantified using Western blot (Fig. 1A). The relative band intensities after normalisation to the *β*-actin loading control were 0, 10.0, 66.3, and 100 for MDA-MB-468, MDA-MB-231, MDA-MB-231/H2N, and SKBR3, respectively.

The relative number of trastuzumab binding sites on each cell line was determined by saturation binding assays using [^111^In]In-trastuzumab in in vitro experiments. This revealed B_max_ values of 1973 ± 135, 18,662 ± 1335, 47,473 ± 1926, and 368,644 ± 6385 cpm for MDA-MB-468, MDA-MB-231, MDA-MB-231/H2N, and SKBR3, respectively (Fig. 1B). K_D_ was quantified as 2.8 ± 0.23 nM.

An immunoreactive fraction of 0.96 ± 0.03 was determined for [^89^Zr]Zr-trastuzumab, the main imaging agent used for in vivo imaging. This result indicates that only a negligible degree of disruption to antigen binding properties occurred as a result of the DFO-modification and radiolabelling procedures (Fig. S2).

### 3.2 Dual-isotope imaging and ex vivo biodistribution analysis

The ability of the MILabs VECTor^4^ imaging system to simultaneously acquire images for ^111^In and ^89^Zr was evaluated using phantoms containing mixtures of known amounts of either radionuclide. Samples containing ^89^Zr only did not show any signal in the constructed ^111^In image, or vice versa. Quantification of ^111^In or ^89^Zr was not influenced by the presence of the other isotope (Fig. S1), corroborating earlier reports on dual-isotope imaging with this system.

#### 3.2.1 MDA-MB-468 (HER2-negative)

**Fig. 2:**
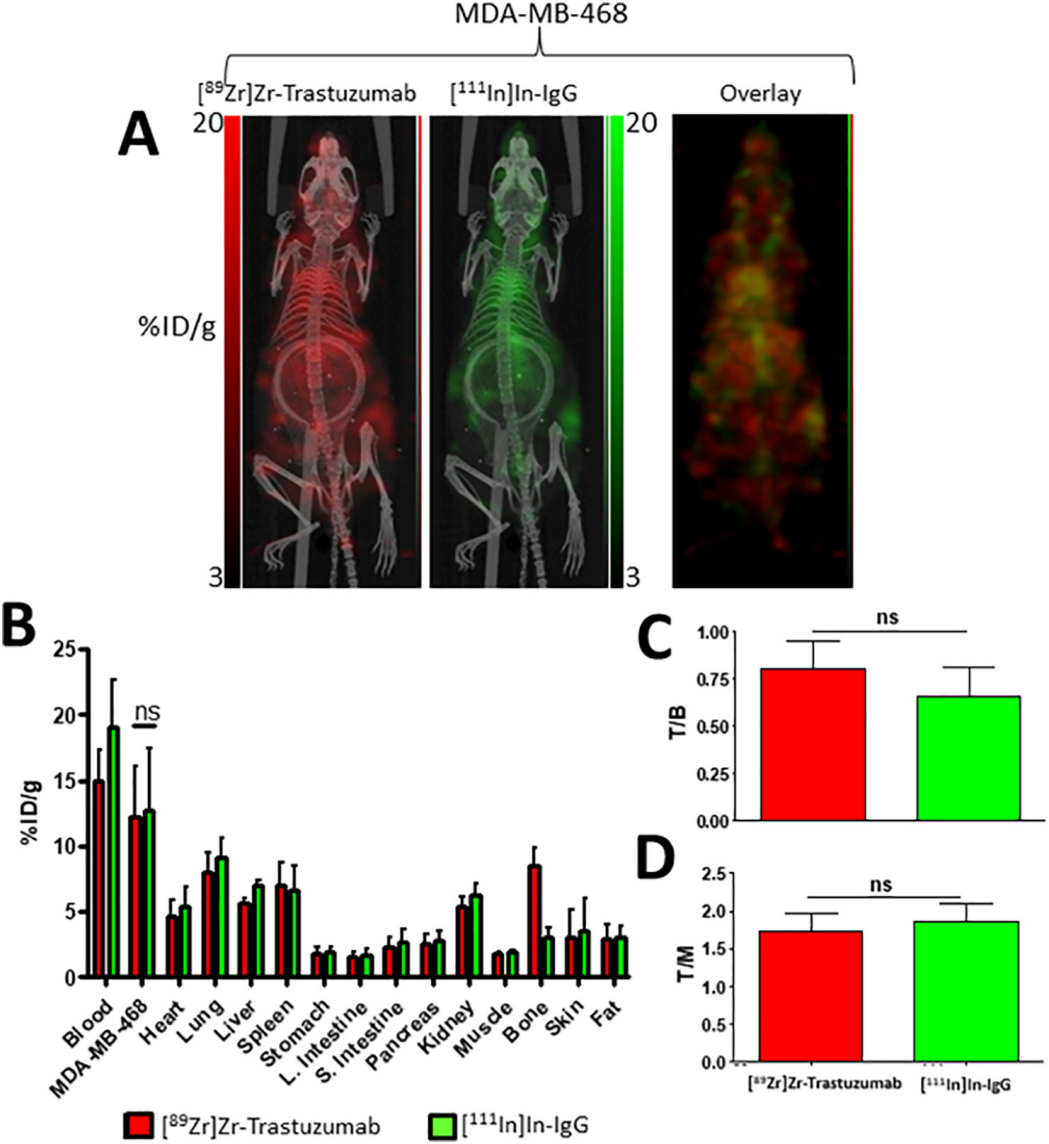
(A) PET/SPECT images of mice bearing MDA-MB-468 tumours at 3 days post injection of [^89^Zr]Zr-trastuzumab and [^111^In]In-IgG. (B) Biodistribution data showing uptake (%ID/g) of each radiolabelled agent in tumours and selected organs. (C) Tumour-to-blood ratios and (D) tumour-to-muscle ratios for each radiolabelled agent. (ns: no statistical significance).

Representative dual-isotope images of mice bearing MDA-MB-468 xenograft tumours (Fig. 2A) and biodistribution data (Fig. 2B) revealed that the co-administered antibodies have a highly comparable overall biodistribution. Analysis of the biodistribution data indicated that the uptake of [^89^Zr]Zr-trastuzumab and [^111^In]In-IgG in the HER2-negative tumours was statistically indistinguishable (12.2 ± 3.8 and 12.7 ± 4.7%ID/g, respectively; P > 0.05). The only significant difference was in bone which had higher ^89^Zr uptake compared to ^111^In (8.5 ± 1.4 and 3.1 ± 0.7%ID/g, respectively; P = 0.004). These values are consistent with those from previous studies in mice involving ^89^Zr-DFO- and ^111^In-DTPA-modified antibodies [[26], [27], [28]] and this disparity is attributed to differences in chelate stability and the particularly osteophilic nature of ^89^Zr [29]. Despite this, it remains that the uptake of both radiolabelled agents in the tumours and all of the other selected organs was not statistically different (Fig. 2B, Table S1; P > 0.05), indicating that the degree of dissociated ^89^Zr did not significantly alter the overall biodistribution compared to the non-specific [^111^In]In-IgG. In addition, the respective tumour-to-blood (T/B; Fig. 2C) and tumour-to-muscle (T/M; Fig. 2D) ratios for [^89^Zr]Zr-trastuzumab (0.8 ± 0.1 and 7.1 ± 2.4, respectively) and [^111^In]In-IgG (0.7 ± 0.2 and 6.8 ± 2.5, respectively) were also similar (P > 0.05).

Accordingly, the [^89^Zr]Zr-trastuzumab: [^111^In]In-IgG uptake ratio in these HER2-negative tumours was 1.0 ± 0.1 and this was in close agreement with volume-of-interest analysis of the reconstructed images which yielded a ratio of 1.3 ± 0.3 (P > 0.05). This corroboration between imaging analysis and ex vivo biodistribution data was found for all the tumour models used in this study (Fig. S3).

#### 3.2.2 MDA-MB-231 (low-HER2) and MDA-MB-231/H2N (medium-HER2)

**Fig. 3:**
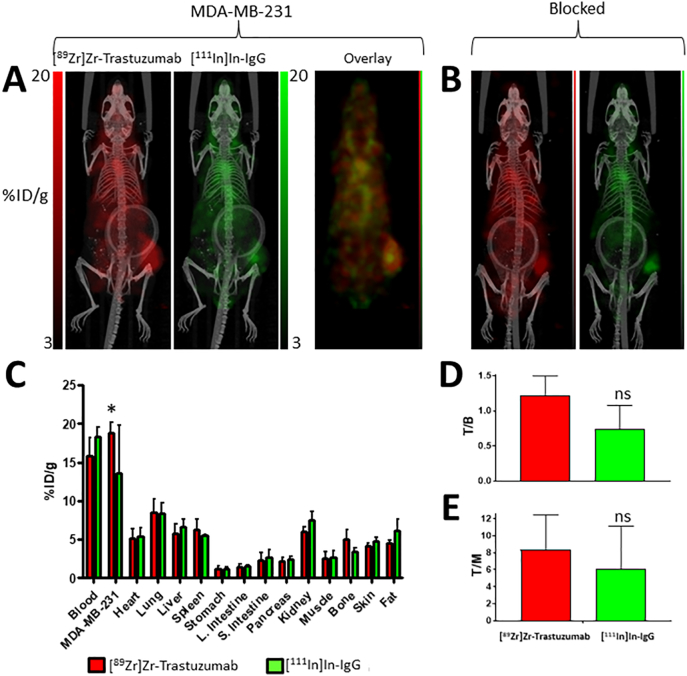
(A) PET/SPECT images of mice bearing MDA-MB-231 tumours at 3 days post injection of [^89^Zr]Zr-trastuzumab and [^111^In]In-IgG. (B) PET/SPECT images of mice bearing MDA-MB-231 tumours at 3 days post injection of [^89^Zr]Zr-trastuzumab, [^111^In]In-IgG, and a blocking dose (0.5 mg) of unlabelled trastuzumab. (C) Biodistribution data showing uptake (%ID/g) of each radiolabelled agent in tumours and selected organs. (D) Tumour-to-blood ratios and (E) tumour-to-muscle ratios for each radiolabelled agent. (ns: no statistical significance; *P < 0.05).

**Fig. 4:**
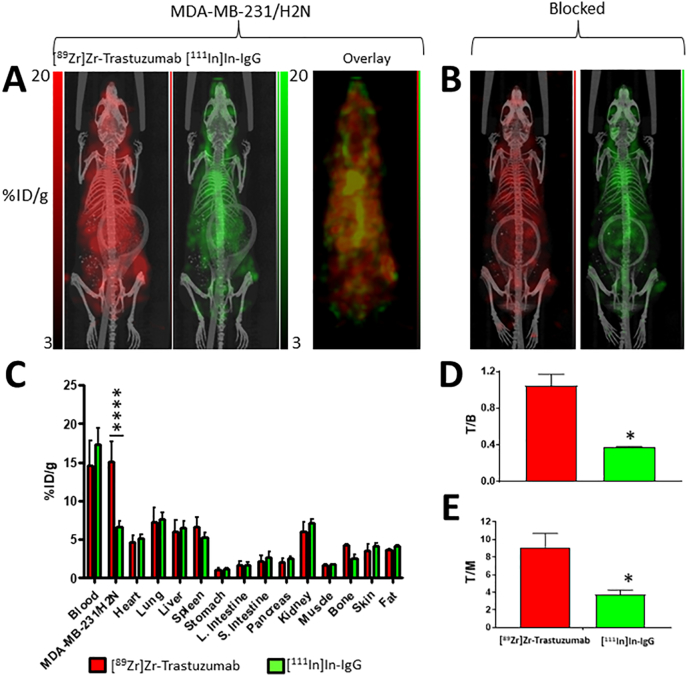
(A) PET/SPECT images of mice bearing MDA-MB-231/H2N tumours at 3 days post injection of [^89^Zr]Zr-trastuzumab and [^111^In]In-IgG. (B) PET/SPECT images of mice bearing MDA-MB-231/H2N tumours at 3 days post injection of [^89^Zr]Zr-trastuzumab, [^111^In]In-IgG, and a blocking dose (0.5 mg) of unlabelled trastuzumab. (C) Biodistribution data showing uptake (%ID/g) of each radiolabelled agent in tumours and selected organs. (D) Tumour-to-blood ratios and (E) tumour-to-muscle ratios for each radiolabelled agent. (*P < 0.05; ****P < 0.0001).

Surprisingly, overall uptake of [^89^Zr]Zr-trastuzumab in the MDA-MB-231 (Fig. 3A–B) and MDA-MB-231/H2N (Fig. 4A–B) tumours was not significantly different (18.8 ± 1.4 and 15.1 ± 2.6%ID/g, respectively; P > 0.05), despite the cell lines having different HER2 expression levels (in vitro B_max_ values were 18,662 ± 1335 and 47,473 ± 1926 cpm, respectively). Similarly, T/B ratios were not different for [^89^Zr]Zr-trastuzumab (1.1 ± 0.1 and 1.0 ± 0.1, respectively; P > 0.05; Figs. 3D, 4D). The T/M ratio was lower, however, for the MDA-MB-231 tumour model compared to the MDA-MB-231/H2N model (8.41 ± 4.05 and 9.1 ± 1.6, respectively; P < 0.05; Figs. 3E, 4E). The reason for the unexpected lack of difference in tumour uptake and T/B ratios became clear when the non-specific uptake of the control IgG was taken into consideration. MDA-MB-231 tumours tended to show higher non-specific uptake (13.5 ± 6.2%ID/g) compared to MDA-MB-231/H2N (6.6 ± 0.8%ID/g; P < 0.10) (Figs. 3C, 4C). Accordingly, the [^89^Zr]Zr-trastuzumab: [^111^In]In-IgG uptake ratios were 1.5 ± 0.5 and 2.3 ± 0.2, respectively (P = 0.31). In mice co-administered a blocking dose (0.5 mg) of unlabelled trastuzumab, a reduction of HER2-mediated [^89^Zr]Zr-trastuzumab uptake was observed, resulting in intratumoural ^89^Zr:^111^In ratios of 0.9 ± 0.1 and 1.4 ± 0.1 for MDA-MB-231 and MDA-MB-231/H2N tumour-bearing mice, respectively. Blocking results in a ratio between [^89^Zr]Zr-trastuzumab vs. [^111^In]In-IgG uptake in the tumour that was not statistically significant from the theoretical value of 1 for MDA-MB-231 xenografts (P = 0.42), but not for MDA-MB-231/H2N tumours (P = 0.008).

It is worth noting that in these two tumour models, the alternative radioisotope combination (i.e. [^111^In]In-trastuzumab/^89^Zr-IgG) was also evaluated. In this case, uptake ratios in the tumours and all of the selected organs were not statistically different to those obtained with [^89^Zr]Zr-trastuzumab: [^111^In]In-IgG (P > 0.05; Fig. S4), indicating that the selection of radioisotope combination is inconsequential. In addition, to determine any influence of additional IgG dose on the biodistribution of the radiolabelled antibody, uptake of [^89^Zr]Zr-trastuzumab with and without co-injection of ^111^In-labelled or non-radiolabelled non-specific IgG control was quantified. The uptake of [^89^Zr]Zr-trastuzumab was not significantly altered (P > 0.05) in MDA-MB-231/H2N xenografts, nor in any of the normal tissues, after injection of isotype control antibody suggesting the normalisation technique employed here can be employed without affecting the original measurement.

#### 3.2.3 SKBR3 (high-HER2)

**Fig. 5:**
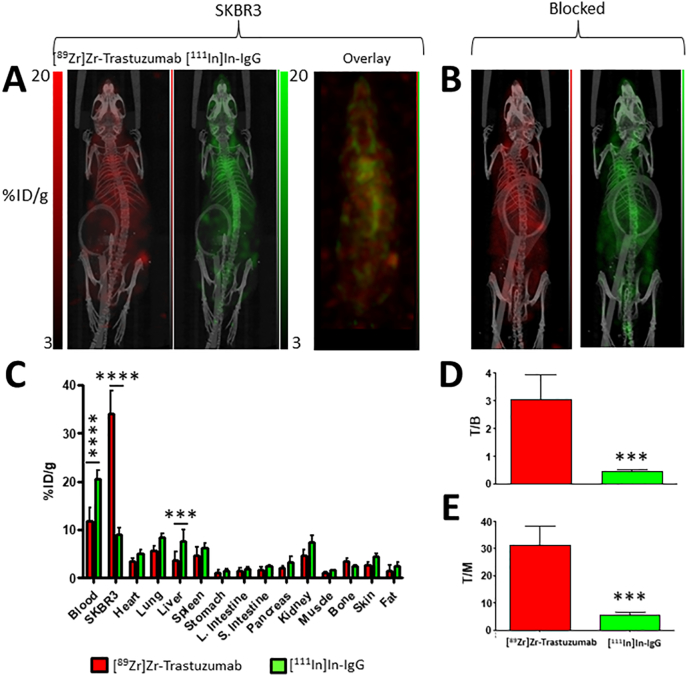
(A) PET/SPECT images of mice bearing SKBR3 tumours at 3 days post injection of [^89^Zr]Zr-trastuzumab and [^111^In]In-IgG. (B) PET/SPECT images of mice bearing SKBR3 tumours at 3 days post injection of [^89^Zr]Zr-trastuzumab, [^111^In]In-IgG, and a blocking dose (0.5 mg) of unlabelled trastuzumab. (C) Biodistribution data showing uptake (%ID/g) of each radiolabelled agent in tumours and selected organs. (D) Tumour-to-blood ratios and (E) tumour-to-muscle ratios for each radiolabelled agent. (***P < 0.001; ****P < 0.0001).

**Fig. 6:**
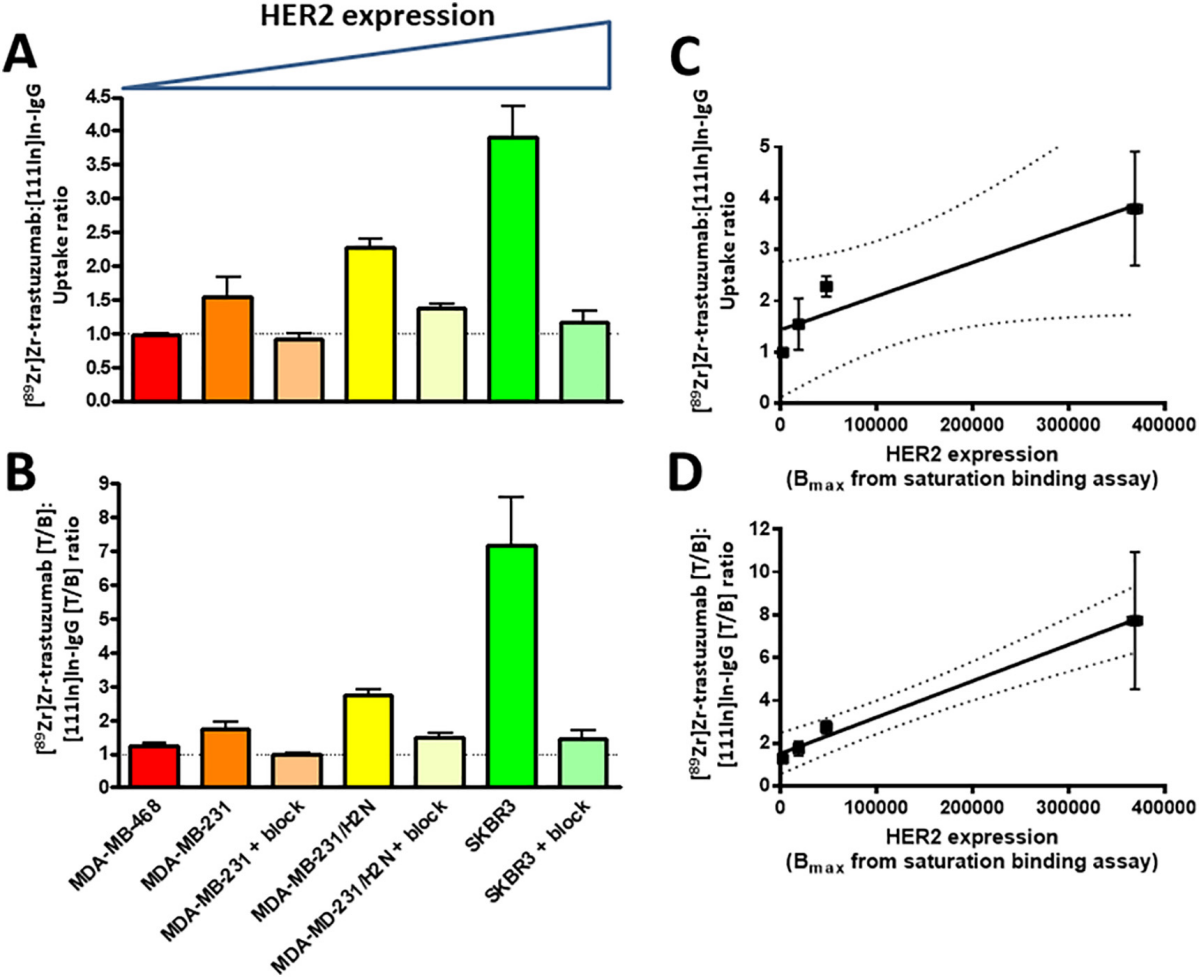
(A) The [^89^Zr]Zr-trastuzumab/[^111^In]In-IgG uptake ratios and (B) the [^89^Zr]Zr-trastuzumab T/B: [^111^In]In-IgG T/B ratios for each of the tumour models used in the study. (C) Correlation between overall uptake of [^89^Zr]Zr-trastuzumab/[^111^In]In-IgG and expression levels of HER2 determined by saturation binding assay. (D) Correlation between [^89^Zr]Zr-trastuzumab T/B: [^111^In]In-IgG T/B ratios and expression levels of HER2 determined by saturation binding assay. Error bars for (A) and (B) represent standard error of the mean.

As expected, the uptake of [^89^Zr]Zr-trastuzumab in SKBR3 tumours was higher compared to each of the other tumour models (34.1 ± 4.8%ID/g; P < 0.006; Figs. 5A–B and S5). Similarly, higher T/B (Fig. 5C) and T/M (Fig. 5D) ratios were also obtained (3.0 ± 0.9 [P < 0.002] and 31.0 ± 7.1 [P < 0.002], respectively). Furthermore, the ratio of intratumoural ^89^Zr:^111^In (3.9 ± 0.9) was significantly higher than each of the other tumour types (P < 0.007; Fig. 6A).

In contrast with the other tumour models used in this study, the respective biodistribution profiles of [^89^Zr]Zr-trastuzumab and [^111^In]In-IgG in SKBR3 tumour-bearing mice were markedly different as significant differences in uptake (%ID/g) were observed for most organs (Fig. 5B). In this case, the considerable extent of HER2-mediated sequestration of [^89^Zr]Zr-trastuzumab to the tumour was likely responsible for these differences. This sequestration effect alters the input function (i.e. the concentration of radiolabelled antibody in blood plasma as a function of time) of [^89^Zr]Zr-trastuzumab compared to [^111^In]In-IgG and this therefore presents an important variable factor which requires consideration. Accordingly, the amount of [^89^Zr]Zr-trastuzumab in blood (%ID/g) at 72 h post injection was selected as the most relevant normalisation parameter to reflect this disparity, and thus the T/B ratios for [^89^Zr]Zr-trastuzumab and [^111^In]In-IgG for all tumour groups were compared (Fig. 6B). As a result of this analysis, a comparison of [^89^Zr]Zr-trastuzumab (T/B): [^111^In]In-IgG (T/B) ratios against the HER2 expression levels (B_max_) for each tumour type, which also revealed a strong correlation with empirically determined HER2 expression levels than [^89^Zr]Zr-trastuzumab: [^111^In]In-IgG tumour uptake ratios alone (R = 0.995, P = 0.0025 vs. R = 0.94, P = 0.029, respectively; Fig. 6A–D). The conventional un-normalised [^89^Zr]Zr-trastuzumab uptake (%ID/g) values were poorly correlated with HER2 expression levels (R = 0.92, P = 0.04, Fig. S6). Voxel-per-voxel subtraction of the non-specific signal also correlates well with the HER2-expresison levels B_max_ values for the tumour models used in this study (R = 0.97, P = 0.030; Fig. S7).

#### 3.2.4 Visualising HER2-mediated uptake of [^89^Zr]Zr-trastuzumab

**Fig. 7:**
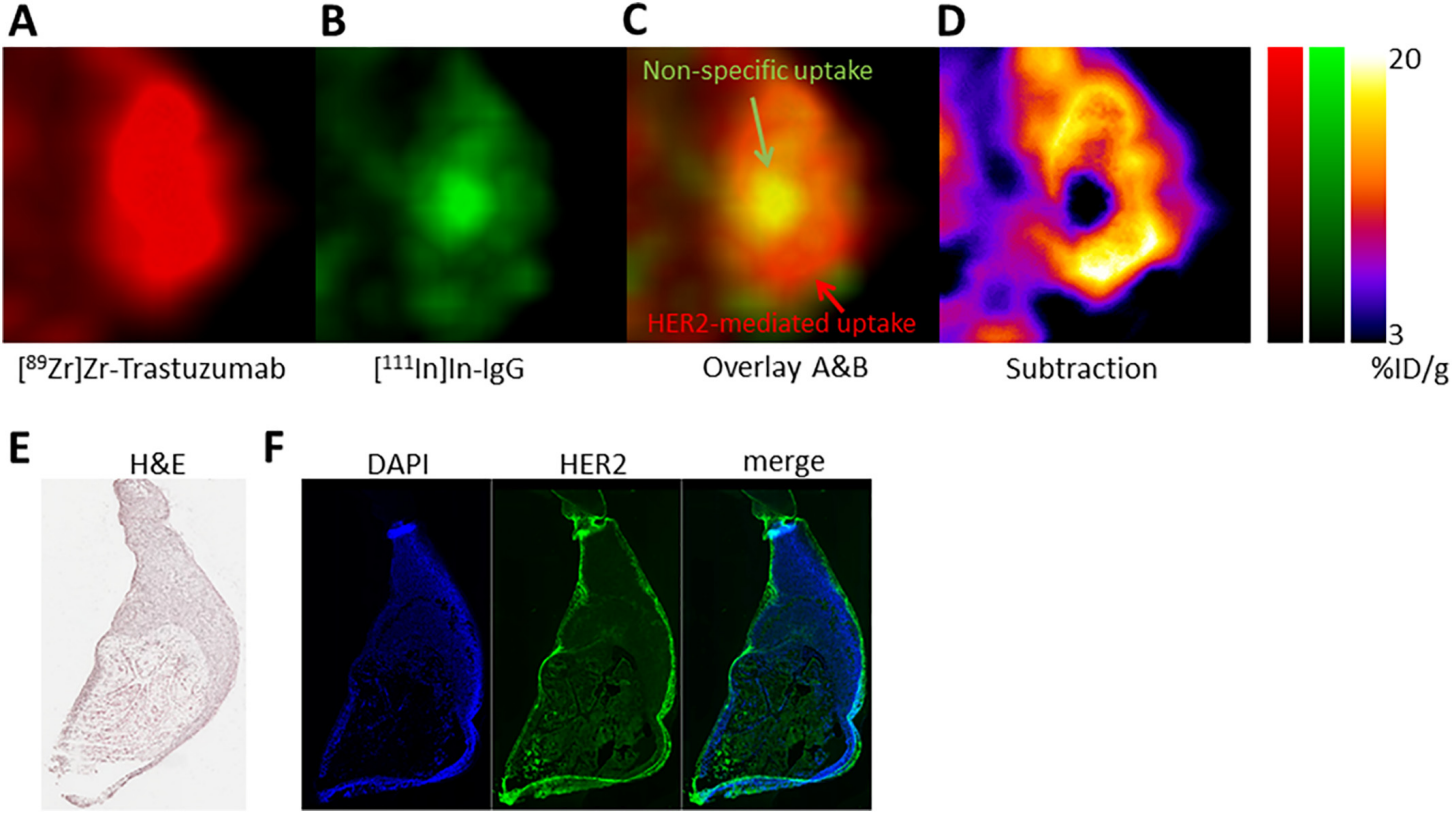
(A) Magnified sections of a representative tumour (MDA-MB-231) showing differences in the intratumoural distribution of [^89^Zr]Zr-trastuzumab (A) and [^111^In]In-IgG (B). Images overlaid in (C). (D) Image resulting from subtraction of the [^111^In]In-IgG signal from the overlaid image showing the HER2-specific uptake of [^89^Zr]Zr-trastuzumab. (E–F) H&E staining and immunohistochemistry staining HER2 in adjacent sections (blue: DAPI; green: HER2).

Dual isotope imaging with SPECT, or indeed performing of PET and SPECT simultaneously has been reported before in the literature [[12], [13], [14], [15], [16], [17]]. However, combining a radiolabelled antibody with its own negative control, and normalising for non-specific uptake to look at intratumoural heterogeneity of epitope-meditated uptake has, to the best of our knowledge, not yet been described (Fig. 7A–E). An example of heterogeneous uptake of [^89^Zr]Zr-trastuzumab (i.e. HER2-mediated and passive accumulation) in a representative low HER-2 expressing MDA-MB-231 tumour can be observed in the magnified image shown in Fig. 7A (red). In an identical view of the same area, non-specific uptake of control IgG (Fig. 7B; green) can been observed pooling in a central compartment (a necrotic region, confirmed by H&E staining, Fig. 7E) within the tumour. The overlaid images (Fig. 7C) and the image which results from subtraction of the non-specific signal from the [^89^Zr]Zr-trastuzumab image (Fig. 7D) each reveal the total absence of HER2-mediated uptake of [^89^Zr]Zr-trastuzumab within this central compartment.

## 4 Discussion

Here, we demonstrated that molecular imaging using dual isotope imaging with antibodies is a potent tool that allows quantification of total tumour epitope-specific uptake of the antibody and correlates well with epitope expression levels. More strikingly, we showed that detailed intratumoural heterogeneity of epitope expression correlates well with background-corrected antibody images – at least in the tumour models we tested here.

For the current study, we used a MILabs VECTOR4 imaging camera, which allows simultaneous SPECT acquisition of a range of radionuclides, from ‘classical’ SPECT isotopes such as ^99m^Tc and ^111^In, to positron emitter such as ^18^F and ^89^Zr, using a set of pinholes [30]. Multi-isotope imaging has been described previously [31]. Therefore, no co-registration between SPECT and PET images was necessary. As such, the basis of the technique we have used here is not new. In fact, dual isotope imaging using SPECT with radiolabelled antibodies has been used since the 1980's [32], and dual isotope SPECT imaging has been used to e.g. visualise the fate of different parts of therapeutic nanoparticles [33]. Non-specific uptake of antibodies, radiolabelled or otherwise, has been known for longer than that. It should be said that molecular imaging using smaller compounds, such as radiolabelled small molecules, peptides, or antibody fragments, engineered antibody-derived proteins such as diabodies, minibodies, affibodies and nanobodies, overcome the issues associated with non-specific uptake of larger molecules. Nonetheless, because of their superior affinity and selectivity, antibodies continue to be used as the basis for imaging agents, and radiolabelled antibodies are being used to study the in vivo pharmacokinetics and pharmacodynamics of immunotherapies and antibody-drug conjugates [34].

What dual isotope imaging allowed us to do, is to compensate, on a voxel-per-voxel basis, for the non-specific component of antibody uptake in the tumour. This enabled us to build a detailed map of epitope-specific uptake heterogeneity within the tumour, which correlated well with that same epitope heterogeneity as assessed by immunohistochemistry.

A key limitation of the methodology used here is the inherent difference in partial volume effects between ^111^In and ^89^Zr on the voxel scale. Here, we addressed this in first approximation by reconstruction of the ^111^In signal to a lower resolution than possible using the MILabs Vector imaging system, artificially lowering the resolution of the ^111^In signal to a similar level of that of the ^89^Zr signal. Additional work is needed to address this challenge and allow true quantitative subtraction imaging.

We postulate that the technique used here will be especially useful for antibody-mediated imaging and quantifying lowly abundant targets such as HER3, or intracellular targets such as γH2AX, where the epitope-specific part of total tumour uptake is relatively low compared to non-specific uptake (as exemplified here in the case of the MDA-MB-231/H2N). Dual isotope imaging will therefore also be useful for the evaluation of experimental tumour therapies [35] to guide e.g. radiotherapy dose painting [36] in preclinical models, or to study in detail the delivery of antibody-drug conjugates [37]. Another advantage is the halving of the number of animals for therapy evaluation studies. For now, we have only evaluated dual isotope imaging in preclinical models. Modern SPECT cameras for patient use are able to quantify images, but currently this approach may be limited to non-positron emitting radioisotopes, and the financial cost of administering two radiolabelled antibodies may prove prohibitive to translate the technique to patients for routine imaging. The additional radiation exposure associated with concurrent administration of two imaging agents is also a disadvantage. In addition, one of the main arguments for non-specific uptake of macromolecular compounds in tumour tissue, the enhanced perfusion and retention effect (EPR), may not be as relevant in human patients than it is in rodents [38,39].

Our results certainly lead to questions regarding the non-specific uptake of radiolabelled antibodies in tumour models. Why should non-specific uptake be so different between what are, to all intents and purposes, nearly identical models, the MDA-MB-231 and MDA-MB-231/H2N xenografts, that are genetically identical apart from knock-in of the HER2 receptor in the latter. Indeed, uptake of the non-specific antibody was 6.6%ID/g in the one, versus 13.5%ID/g in the other. Reasons may include (1) differences in vascular density, vascular architecture and fenestration; (2) differences in lymphatic drainage; and (3) differences in the EPR effect that may be further enhanced by changes in other phenotypic factors such as, bradykinin, nitric oxide, prostaglandins, vascular endothelial growth factor, or tumour necrosis factor and others. It is of note that the dual-isotope-based normalisation technique described here of course takes into account all the inter-individual differences in tumour vascularisation.

## 5 Conclusions

The dual-isotope method described herein provides a quantitative and highly personalised value of epitope-specific uptake for antibody-based radiopharmaceuticals. Normalisation of [^89^Zr]Zr-trastuzumab uptake and tumour-to-blood contrast ratios to corresponding values obtained with [^111^In]In-IgG resulted in metrics strongly correlated with empirically determined HER2 expression levels. Furthermore, subtraction of the non-specific [^111^In]In-IgG signal from the [^89^Zr]Zr-trastuzumab image reveals intratumoural regions of non-specific uptake which otherwise would remain obscured. This approach also offers utility in preclinical research and development studies based on examining drug penetration, target engagement, tumour heterogeneity, radiation dose painting, and beyond.

## Appendix A Supplementary data

Supplementary material Image 1
