# Supplementary material for: Dual-isotope imaging allows in vivo immunohistochemistry using radiolabelled antibodies in tumours
Source: Nucl Med Biol. 2019 Mar;70:14–22. doi: 10.1016/j.nucmedbio.2019.01.010 (PMC6599172; doi:10.1016/j.nucmedbio.2019.01.010)
Supplement: Supplementary file 1 — Supplementary material [file mmc1.docx]

**Supporting information for**

**Dual-Isotope Imaging Reveals Inter-Individual Differences in Passive**

**Uptake of Radiolabelled Antibodies in Tumours**

James C. Knight^1^, Michael J. Mosley^1^, Veerle Kersemans^1^, Gemma M. Dias^1^, P. Danny Allen^1^, Sean Smart^1^, and Bart Cornelissen^1^

^1^ CR-UK/MRC Oxford Institute for Radiation Oncology, Department of Oncology, University of Oxford, Oxford, United Kingdom

bart.cornelissen@oncology.ox.ac.uk

**Supplemental Methods**

**PET/SPECT/CT Imaging**

*General*

PET/SPECT/CT imaging was performed using a VECTor^4^ imaging system (MILabs) using a rat collimator with 1.8 mm pinholes. List-mode PET/SPECT data were acquired for 60 minutes and a CT image was then subsequently acquired (55 kV, 0.19 mA) on the same system. For the indium-111 SPECT images, reconstructions were performed using a γ-ray energy window of 156–190 keV (background weight 2.5), 0.8 mm^3^ voxels, 8 subsets, and 6 iterations using the manufacturer’s POSEM reconstruction type. For the zirconium-89 PET images, reconstructions were performed using energy windows of 450-600 keV and 800-1030 keV (background weight 1.2), 0.8 mm^3^ voxels, 128 subsets, and 1 iteration using the manufacturer’s SROSEM reconstruction type. To allow accurate scaling and quantification, calibration factors (determined from phantoms) were applied to the data. PET and SPECT images were each registered to CT and then attenuation corrected. During each imaging session, mice were kept under anaesthesia by inhalation of 2% isofluorane in air and maintained at 37°C. Images were processed and volume-of-interest analyses were performed using the PMod software package (version 3.807, PMOD Technologies).

**Western blotting**

Tumour xenograft tissues were used to prepare total cell lysates by homogenization with RIPA buffer (50 mM Tris pH 8, 1% NP40 substitute, 0.5% sodium deoxycholate, 0.1% SDS, 150 mM NaCl, plus 1x cOMPLETE protease inhibitor cocktail tablet). Protein yields were confirmed with a bicinchoninic acid (BCA) protein assay (Thermo Scientific). Thirty-five micrograms of protein from each tumour xenograft lysate were run on either a NuPAGE Novex 3-8% Tris-Acetate protein gel (for identification of HER2), or a NuPage Novex 4-12% Bis-Tris protein gel (Life Technologies) for identification of the loading-control, *β*-actin. The gel-separated proteins were transferred to PVDF membranes using a Mini-PROTEAN system (Bio-Rad). Non-specific binding sites were blocked by incubation with 5% milk and the blots probed for HER2 expression using a rabbit monoclonal antibody (29D8-2165, Cell Signalling Technology) at 1:1,000 dilution, or *β*-actin expression using a rabbit polyclonal antibody (ab8227, Abcam) at 1:3,000 dilution. Target proteins (HER2 at 185kDa and β-actin at 42 kDa) were visualized using a horseradish peroxidase secondary antibody (Thermo Scientific, catalogue no. 65-6120) at 1:5,000 dilution and subsequent exposure to x-ray film.

**Figure S1:** (A) A representative series of five radioactive phantoms (1-5). Phantom 1 only contains indium-111, phantom 5 only contains zirconium-89. Phantoms 2-4 contain mixtures of indium-111 and zirconium-89 (units: MBq). (B and C) Bar charts showing measured amounts of radioactivity in each phantom (from dose calibrator) and the corresponding values measured from VOI analysis. (D and E) Linear regression analysis of the radioactivity values measured on the dose calibrator and from VOI analysis.

**
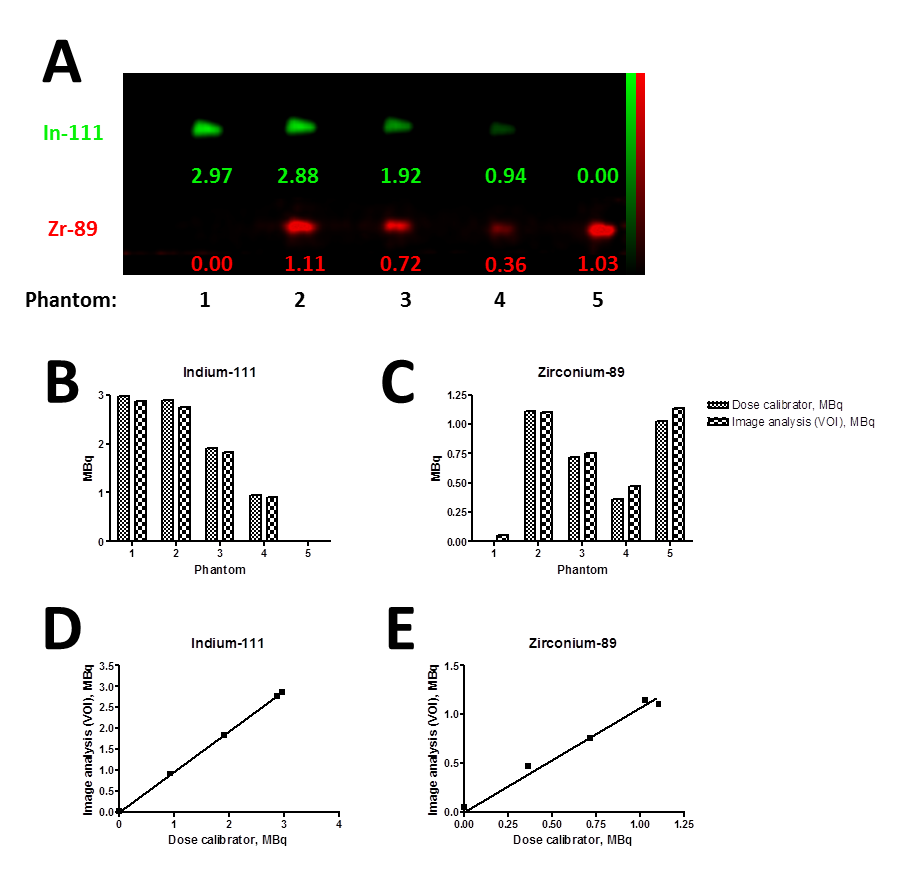
**

**Figure S2:** Determination of immunoreactive fraction of [^89^Zr]Zr-trastuzumab on MDA-MB-231/H2N cells by linear extrapolation to conditions representing an infinite antigen excess.

**Figure S3:** Comparison of tumour uptake (%ID/g) ratios determined by ex vivo γ-counting and VOI analysis of SPECT images.

**Figure S4:** Uptake ratios in MDA-MB-231 tumour bearing mice for each of the two possible radioisotope combinations.

**Figure S5:** Inter-individual differences in uptake of [^89^Zr]Zr-trastuzumab and [^111^In]In-IgG in mice bearing (A) MDA-MB-468, (B) MDA-MB-231, (C) MDA-MB-231/H2N, and (D) SKBR3 tumours.


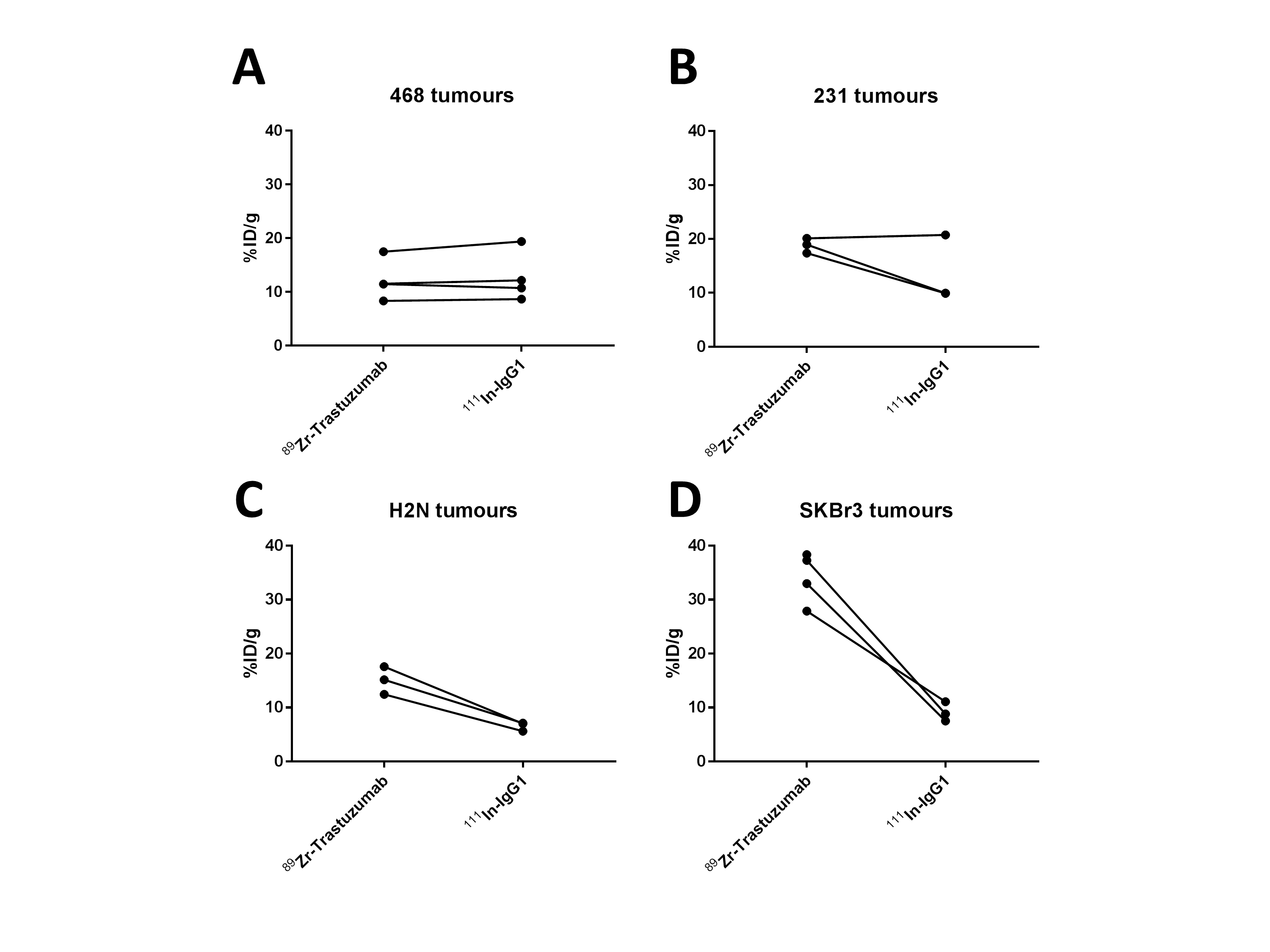


**Figure S6:** Correlation between [^89^Zr]Zr-trastuzumab tumour uptake values and expression levels of HER2 determined by saturation binding assay.

**Figure S7:** Subtraction of [^111^In]In-IgG uptake values (%ID/g) from corresponding uptake values obtained with [^89^Zr]Zr-trastuzumab for each of the tumour types used in the study (left). Correlation of the resulting values with expression levels of HER2 determined by saturation binding assay (right).

**
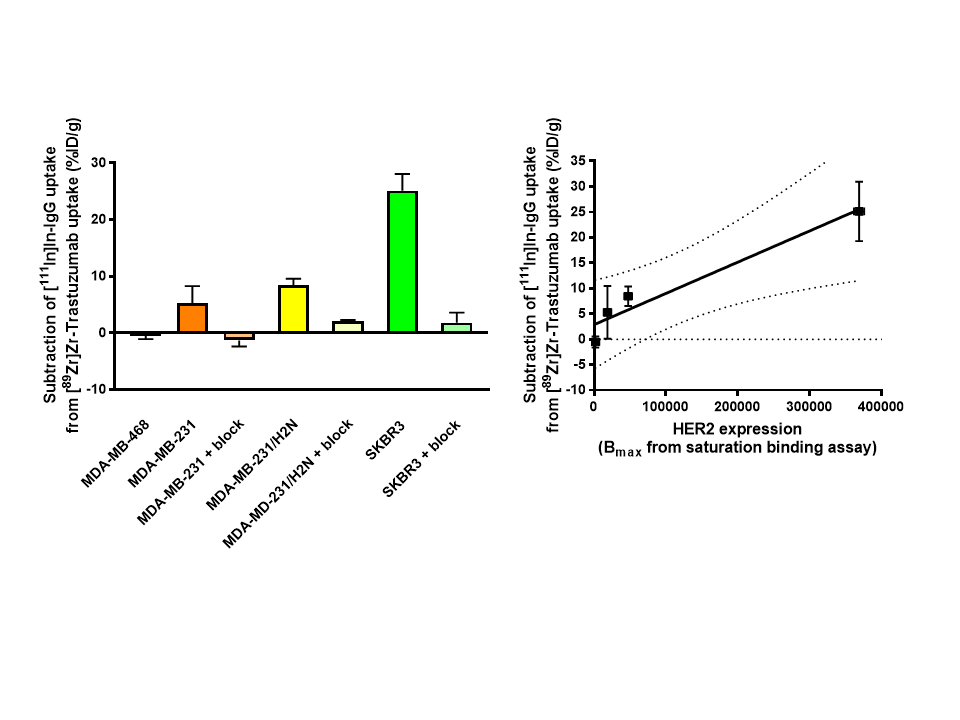
**

**TABLE S1:** *Ex vivo* biodistribution data acquired at 72 h p.i. of [^89^Zr]Zr-trastuzumab and [^111^In]In-IgG in MDA-MB-468 tumour-bearing mice. Values are %ID/g ± SD.

|  | [^89^Zr]Zr-trastuzumab  (*n*=4) | [^111^In]In-IgG  (*n*=4) |
| --- | --- | --- |
| Blood | 14.90±2.34 | 19.07±3.61 |
| Tumour | 12.20±3.83 | 12.72±4.67 |
| Heart | 4.63±1.27 | 5.36±1.46 |
| Lung | 7.97±1.55 | 9.06±1.49 |
| Liver | 5.57±0.46 | 6.95±0.46 |
| Spleen | 6.96±1.76 | 6.64±1.89 |
| Stomach | 1.81±0.48 | 1.87±0.36 |
| Large intestine | 1.53±0.42 | 1.62±0.52 |
| Small intestine | 2.23±0.82 | 2.61±1.01 |
| Pancreas | 2.47±0.80 | 2.81±0.76 |
| Kidney | 5.40±0.75 | 6.19±0.92 |
| Muscle | 1.74±0.22 | 1.86±0.23 |
| Bone | 8.50±1.39 | 3.08±0.73 |
| Skin | 3.04±2.04 | 3.58±2.41 |
| Fat | 2.95±1.11 | 2.99±0.93 |

**TABLE S2:** *Ex vivo* biodistribution data acquired at 72 h p.i. of [^89^Zr]Zr-trastuzumab and [^111^In]In-IgG in MDA-MB-231 tumour-bearing mice. Values are %ID/g ± SD.

|  | [^89^Zr]Zr-trastuzumab  (*n*=3) | [^111^In]In-IgG  (*n*=3) | [^89^Zr]Zr-trastuzumab  + 0.5 mg of trastuzumab (*n*=3) | [^111^In]In-IgG  + 0.5 mg of trastuzumab (*n*=3) |
| --- | --- | --- | --- | --- |
| Blood | 15.81±2.35 | 18.32±1.22 | 18.58±1.11 | 19.83±0.89 |
| Tumour | 18.82±1.38 | 13.53±6.25 | 15.52±11.48 | 16.83±12.60 |
| Heart | 5.16±1.28 | 5.38±1.12 | 5.73±0.83 | 5.75±0.97 |
| Lung | 8.43±1.79 | 8.38±1.31 | 9.72±0.63 | 9.83±0.26 |
| Liver | 5.76±1.22 | 6.67±0.92 | 6.90±1.09 | 6.86±0.62 |
| Spleen | 6.23±1.37 | 5.46±0.14 | 7.92±0.31 | 7.38±0.60 |
| Stomach | 1.16±0.34 | 1.14±0.31 | 2.15±0.55 | 1.98±0.36 |
| Large intestine | 1.47±0.36 | 1.56±0.08 | 2.08±0.04 | 2.05±0.10 |
| Small intestine | 2.30±0.95 | 2.61±1.02 | 2.51±0.16 | 2.62±0.20 |
| Pancreas | 2.17±0.48 | 2.36±0.42 | 2.56±0.42 | 2.94±0.54 |
| Kidney | 6.02±0.65 | 7.45±1.12 | 6.15±0.74 | 6.82±0.76 |
| Muscle | 2.51±0.84 | 2.67±0.80 | 2.33±1.04 | 2.45±1.05 |
| Bone | 4.99±1.28 | 3.38±0.55 | 5.83±0.75 | 3.53±0.25 |
| Skin | 4.20±0.33 | 4.77±0.50 | 4.99±0.70 | 5.73±1.17 |
| Fat | 4.57±0.36 | 6.09±1.57 | 3.58±0.89 | 3.46±0.88 |

**TABLE S3:** *Ex vivo* biodistribution data acquired at 72 h p.i. of [^89^Zr]Zr-trastuzumab and [^111^In]In-IgG in MDA-MB-231/H2N tumour-bearing mice. Values are %ID/g ± SD.

|  | [^89^Zr]Zr-trastuzumab  (*n*=3) | [^111^In]In-IgG  (*n*=3) | [^89^Zr]Zr-trastuzumab  + 0.5 mg of trastuzumab (*n*=4) | [^111^In]In-IgG  + 0.5 mg of trastuzumab (*n*=4) |
| --- | --- | --- | --- | --- |
| Blood | 14.53±3.26 | 17.27±2.15 | 13.81±1.57 | 15.39±3.98 |
| Tumour | 15.05±2.57 | 6.58±0.84 | 7.96±0.75 | 5.84±1.03 |
| Heart | 4.67±0.78 | 5.11±0.58 | 4.02±0.82 | 4.59±0.82 |
| Lung | 7.23±1.90 | 7.64±0.80 | 7.57±0.90 | 8.45±0.97 |
| Liver | 6.03±1.45 | 6.47±0.87 | 5.87±1.74 | 6.18±0.72 |
| Spleen | 6.59±1.30 | 5.29±0.63 | 5.62±1.15 | 5.15±1.05 |
| Stomach | 1.05±0.29 | 1.11±0.13 | 1.09±0.30 | 1.11±0.28 |
| Large intestine | 1.64±0.46 | 1.71±0.30 | 1.53±0.08 | 1.68±0.14 |
| Small intestine | 2.22±-0.74 | 2.61±0.73 | 1.78±0.16 | 2.09±0.10 |
| Pancreas | 2.00±0.48 | 2.48±0.26 | 2.02±0.36 | 2.51±0.39 |
| Kidney | 6.03±1.19 | 7.11±0.50 | 5.45±0.95 | 6.51±1.16 |
| Muscle | 1.65±0.14 | 1.77±0.04 | 1.33±0.20 | 1.62±0.22 |
| Bone | 4.32±0.05 | 2.57±0.42 | 5.33±1.15 | 3.24±0.73 |
| Skin | 3.49±0.96 | 4.18±0.32 | 3.78±0.94 | 4.46±0.70 |
| Fat | 3.65±0.13 | 4.11±0.20 | 3.61±1.21 | 3.55±0.76 |

**TABLE S4:** *Ex vivo* biodistribution data acquired at 72 h p.i. of [^89^Zr]Zr-trastuzumab and [^111^In]In-IgG in SKBR3 tumour-bearing mice. Values are %ID/g ± SD.

|  | [^89^Zr]Zr-trastuzumab  (*n*=4) | [^111^In]In-IgG  (*n*=4) | [^89^Zr]Zr-trastuzumab  + 0.5 mg of trastuzumab (*n*=3) | [^111^In]In-IgG  + 0.5 mg of trastuzumab (*n*=3) |
| --- | --- | --- | --- | --- |
| Blood | 11.74±2.91 | 20.47±1.92 | 13.85±1.93 | 17.07±1.50 |
| Tumour | 34.13±4.76 | 8.99±1.52 | 12.80±2.88 | 10.98±1.40 |
| Heart | 3.42±0.65 | 5.10±0.65 | 4.26±0.41 | 4.69±0.41 |
| Lung | 5.73±0.90 | 8.37±0.93 | 7.19±1.31 | 7.89±1.30 |
| Liver | 5.73±1.77 | 7.71±2.21 | 6.59±0.52 | 8.83±1.15 |
| Spleen | 3.62±1.71 | 6.17±1.06 | 6.27±0.77 | 5.93±0.46 |
| Stomach | 4.65±0.54 | 1.39±0.49 | 1.61±0.09 | 1.71±0.12 |
| Large intestine | 1.10±0.61 | 1.84±0.46 | 2.17±0.38 | 2.22±0.48 |
| Small intestine | 1.40±0.59 | 2.40±0.19 | 1.99±0.27 | 2.39±0.13 |
| Pancreas | 2.06±0.44 | 3.25±1.14 | 2.10±0.43 | 2.51±0.54 |
| Kidney | 4.57±1.27 | 7.34±1.39 | 5.58±0.20 | 7.52±0.26 |
| Muscle | 1.12±0.20 | 1.64±0.04 | 1.41±0.29 | 1.76±0.20 |
| Bone | 3.44±0.59 | 2.38±0.27 | 3.27±0.33 | 2.35±0.04 |
| Skin | 2.71±0.48 | 4.47±0.48 | 2.74±0.19 | 4.03±0.11 |
| Fat | 1.47±1.20 | 2.50±0.70 | 2.38±0.20 | 2.64±0.30 |
